# Reading or listening to review summaries - which method will produce greater understanding of the key outcomes in a cochrane review?

**DOI:** 10.1186/1745-6215-16-S2-O2

**Published:** 2015-11-16

**Authors:** Lisa Maguire, Mike Clarke, Mark Tully

**Affiliations:** 1 Queen's University Belfast, Belfast, UK; 2 University of Liverpool, Liverpool, UK

## Background

Systematic reviews are key to the dissemination of the findings of clinical trials and many readers might access nothing more than a summary of these reviews. Therefore, it is essential that these summaries are clear, understandable and accessible. We explored whether readers understand key messages without having to read the full review, and if there were differences in understanding between various types of summary, including an audio podcast.

## Methods

We selected four Cochrane Reviews:

• 1. Workplace interventions for reducing sitting at work

• 2. Chewing gum for postoperative recovery of gastrointestinal function

• 3. Surgery for weight loss in adults

• 4. Dance movement therapy for depression

Potential participants were contacted via University and organisational mailing lists. Those who wished to take part were asked to select one of the four reviews. After answering a question about what they thought the key finding of the review would be, they were randomly assigned one of four summaries of the review: abstract, plain language summary, audio podcast or transcript of the podcast. They were asked to spend no more than 15 minutes reading or listening to the summary, before answering again the question about the key findings and to indicate whether they would now want to read the full Cochrane Review.

## Results

This research is currently underway and final results will be presented at the conference.

## Conclusion

This study repeats our previous SWAR -2 study with a new set of reviews, which suggested better understanding with the audio podcast.

